# Is Memantine Effective as an NMDA Receptor Antagonist in Adjunctive Therapy for Schizophrenia?

**DOI:** 10.3390/biom10081134

**Published:** 2020-07-31

**Authors:** Tetsuro Kikuchi

**Affiliations:** New Drug Research Division, Pharmaceutical Business Division, Otsuka Pharmaceutical Co., Ltd., 463-10 Kagasuno, Kawauchi-cho, Tokushima 771-0192, Japan; kikuchite@otsuka.jp; Tel.: +81-88-665-2126

**Keywords:** memantine, NMDA receptors, low affinity, fast off-rate, voltage dependency, fast unblocking, partial trapping, uncompetitive antagonist, schizophrenia, adjunctive therapy

## Abstract

Memantine, an *N*-methyl-d-aspartate (NMDA) receptor antagonist approved for treating Alzheimer’s disease, has a good safety profile and is increasingly being studied for possible use in a variety of non-dementia psychiatric disorders. There is an abundance of basic and clinical data that support the hypothesis that NMDA receptor hypofunction contributes to the pathophysiology of schizophrenia. However, there are numerous randomized, double-blind, placebo-controlled clinical trials showing that add-on treatment with memantine improves negative and cognitive symptoms, particularly the negative symptoms of schizophrenia, indicating that memantine as adjunctive therapy in schizophrenia helps to ameliorate negative symptoms and cognitive deficits. It remains unclear why memantine does not show undesirable central nervous system (CNS) side effects in humans unlike other NMDA receptor antagonists, such as phencyclidine and ketamine. However, the answer could lie in the fact that it would appear that memantine works as a low-affinity, fast off-rate, voltage-dependent, and uncompetitive antagonist with preferential inhibition of extrasynaptic receptors. It is reasonable to assume that the effects of memantine as adjunctive therapy on negative symptoms and cognitive deficits in schizophrenia may derive primarily, if not totally, from its NMDA receptor antagonist activity at NMDA receptors including extrasynaptic receptors in the CNS.

## 1. Introduction

The pathogenesis of schizophrenia remains unestablished to date. However, according to the dopamine hyperactivity theory, hyperactivity of dopaminergic neurotransmission is assumed to play a central role. Thus, in recent years, many types of antipsychotics with actions at dopamine receptors have been introduced into the clinical setting. In fact, most conventional antipsychotics (including D_2_ receptor partial agonists) basically exhibit functional D_2_ receptor antagonist activity [1,2]. However, many schizophrenia patients do not respond completely to treatment with conventional antipsychotics and continue to experience positive and negative symptoms, cognitive impairment, depression, and other symptoms. Theoretically, the residual symptoms, which do not respond completely to treatment with conventional antipsychotics (i.e., those having functional D_2_ receptor antagonist activity), are thought to derive from a non-dopaminergic mechanism. Pharmaceutical drugs with various mechanisms of action have been considered or tried as adjunctive therapy for schizophrenia patients. These drugs include serotonin-reuptake inhibitors, 5-HT_2C_, 5-HT_3_, and 5-HT_6_ receptor antagonists, α-7 nicotinic and M_1_ muscarinic receptor agonists, GABA-A receptor agonists, GABA-B receptor antagonists, anti-inflammatory drugs, cannabinoids, such as cannabidiol, cholecystokinin agonists, neurokinin-3 receptor antagonists, and glutamatergic agents [3]. Memantine is one of the adjunctive therapy options for treating schizophrenia patients. It is an *N*-methyl-d-aspartate (NMDA) receptor antagonist that has been approved for the treatment of moderate to severe Alzheimer’s disease [4]. Articles suggesting that memantine may be an effective adjunctive therapy in schizophrenia have been published. This review examines the past and current information on memantine as adjunctive therapy in schizophrenia and seeks to ascertain if the NMDA receptor antagonist activity of memantine could contribute to adjunctive therapy effects in schizophrenia.

## 2. NMDA Receptor Hypofunction in Schizophrenia

Glutamate is the primary excitatory neurotransmitter and the most abundant neurotransmitter in the brain. Glutamate activates G protein-coupled metabotropic receptors and ionotropic receptors. The ionotropic receptors are divided into three subtypes: kainate receptors, α-amino-3-hydroxy-5-methylisoxaole-4-propionate (AMPA) receptors, and NMDA receptors. Kainate and AMPA receptors play a key role in excitatory neurotransmission by mediating fast postsynaptic potentials. NMDA receptors have a central role in long-term potentiation (LTP), a major form of synaptic plasticity associated with reversible changes in synaptic transmission efficiency [5]. NMDA receptors constitute a heterotetrameric complex of two essential GluN1 subunits with either two GluN2 subunits or a combination of GluN2 and GluN3 subunits [5,6]. For more detailed information on the structure of NMDA receptors, see the review by Uno and Coyle in 2019 [6].

NMDA receptors are essential for coincidence detection. Coincidence detection can be described as the simultaneous presence of two signals. At resting membrane potential, Mg^2+^ blocks the NMDA channel. Postsynaptic membrane depolarization is typically induced by AMPA receptor activation due to glutamate. The depolarization of postsynaptic membrane represents the first signal, caused by removing Mg^2+^ from the binding site of the channel pore. Glutamate, which is released from presynaptic sites, represents the second signal. The binding of glutamate to the ligand-binding domain (LBD) of GluN2 subunits and the binding of glycine or D-serine to the glycine modulatory site (GMS) of GluN1 subunits are required simultaneously to open ion channels of NMDA receptors (Ca^2+^ influx). The Ca^2+^ influx induces a cascade of intracellular events and LTP of synaptic transmission. However, excessively increased glutamate in the synapse leads to overstimulation of NMDA receptors. Thus, Ca^2+^ influx increases greatly, resulting in remarkable elevation of intracellular Ca^2+^ levels. Consequently, toxic metabolic processes that may lead to nerve-cell death are started. Calcium is a key molecule for intracellular signaling that is involved in many types of neuroplasticity, but excessive calcium levels can lead to cell death [5,6].

**Figure 1:**
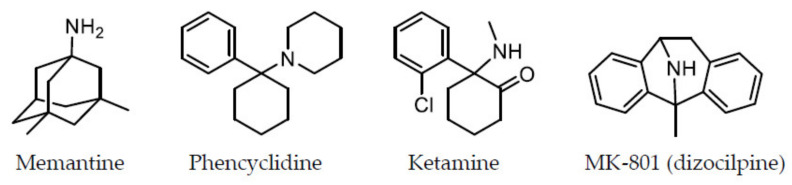
Chemical structures of memantine and other NMDA receptor antagonists.

There is an abundance of data that support the hypothesis that NMDA receptor hypofunction contributes to the pathophysiology of schizophrenia. This NMDA receptor hypofunction hypothesis was derived from clinical findings. Dissociative anesthetic agents, such as phencyclidine (PCP) [7] and ketamine [8], are known to induce schizophrenic-like symptoms with positive and negative symptoms, as well as cognitive impairment, in healthy humans, and ketamine is known to worsen symptoms in patients with schizophrenia [9,10]. It was demonstrated that PCP and ketamine are NMDA receptor antagonists [5,6]. MK-801 (dizocilpine) was originally developed as a more refined compound, and it was considerably more potent than PCP in producing antagonist activity at NMDA receptors [11]. However, MK-801 was not fully studied for human use, because it produced significant mental side effects during its early clinical trials as an anticonvulsant, and it appeared to have the same psychotomimetic properties as PCP in humans based on behavioral studies using a variety of species, including rhesus monkeys [12]. Thus, it was proposed that schizophrenia could result from hypofunction of NMDA receptors [13,14,15]. The chemical structures of memantine and other NMDA receptor antagonists are represented in Figure 1. The hypothesis of NMDA receptor hypofunction in schizophrenia is supported by findings of morphological changes in glutamatergic neuron dendrites of the cerebral cortex in patients with schizophrenia and of decreased levels of synaptophysin, which is an axon bouton marker [16]. An imaging study using the novel NMDA receptor single-photon emission computed tomography (SPECT) tracer, [^123^I]CNS-1261, found the first evidence of reduced hippocampal NMDA receptor binding in medication-free patients with schizophrenia but not in patients receiving antipsychotics compared to healthy volunteers [17]. After the finding, the NMDA receptor hypofunction was supported by ^1^H magnetic resonance spectroscopy and positron emission tomography/SPECT studies [18]. Furthermore, certain genetic-association studies revealed that NMDA receptor function may involve schizophrenia risk genes, such as D-amino acid oxidase, G72, neuregulin 1, and dysbindin [19,20]. Individuals with anti-NMDA receptor encephalitis, which is caused by antibodies against the extracellular epitopes on the amino-terminal domain of the GluN1 subunit, initially exhibit schizophrenia-like symptoms. The antibodies are considered to induce the internalization of postsynaptic NMDA receptor clusters, thereby causing NMDA receptor downregulation to induce the hypofunction [21]. In animals also, some data support the hypothesis that NMDA receptor hypofunction contributes to the pathophysiology of schizophrenia. In pharmacologically induced models, treatment of animals with NMDA receptor antagonists, such as PCP, ketamine, and MK-801, leads to neurochemical, morphological, and cognitive deficits similar to those observed in schizophrenia [5,15]. Studies on genetic animal models have also provided abundant data suggesting that decreased NMDA receptor activity may induce changes in the brain and behavior similar to those reported in schizophrenia [5,15].

Regarding the hypothesis of NMDA receptor hypofunction, it has been proposed that decreased NMDA receptor activity on γ-aminobutyric acid (GABA) interneurons in the prefrontal cortex results in disinhibition of cortical brainstem glutamatergic neurons that project to the ventral tegmental area (VTA). This disinhibition results in excessive glutamatergic tone, which leads to excessive activation of the mesolimbic dopaminergic pathway and produces excessive release of dopamine in the nucleus accumbens, inducing positive symptoms. Similarly, negative and cognitive symptoms may result from NMDA receptor hypofunction. Hyperactivity of cortical brainstem glutamatergic neurons possibly results in excessive activation of GABAergic interneurons in the VTA. Subsequently, the GABAergic interneurons release excessive GABA and induce excessive inhibition of the mesocortical dopaminergic pathway, which then becomes unable to supply an adequate release of dopamine in the prefrontal cortex, inducing negative and cognitive symptoms. Thus, abnormal glutamate neural circuits due to defective NMDA receptors may actually cause excessive dopamine release in the mesolimbic pathway and deficiency of dopamine release in the mesocortical pathway [22].

Based on the NMDA receptor hypofunction hypothesis, many candidate substances with the ability to ameliorate the hypofunction of NMDA receptors have been tried in the clinical setting. It has been reported that glycine, _D_-cycloserine, _D_-serine, sodium benzoate, sarcosine, bitopertin, n-acetylcysteine, mGlu_2/3_ receptor agonists, mGlu_2_ receptor positive allosteric modulators, and mGlu_5_ receptor positive allosteric modulators provide some benefit in terms of clinical outcome in schizophrenics and subjects with prodromal syndrome [6,23]. However, at this stage, none of these drugs have been approved for clinical use in the treatment of schizophrenia.

## 3. Memantine as Adjunctive Therapy in Schizophrenia

There are many reports that the NMDA receptor antagonist memantine improves schizophrenia despite what is implied by the hypothesis of NMDA receptor hypofunction in schizophrenia. Randomized, double-blind, placebo-controlled trials of memantine as adjunctive therapy in schizophrenia have been published starting from around 2009. The first meta-analysis addressing the efficacy and effectiveness of NMDA receptor antagonist adjunctive therapy in schizophrenia was published by Kishi and Iwata in 2013 [24]. They reported that the NMDA receptor antagonists memantine and amantadine as adjunctive therapy may improve cognitive function in patients with schizophrenia [24]. Rezaei et al. conducted a double-blind placebo-controlled clinical trial in patients with schizophrenia stabilized with risperidone. They reported in 2013 that the memantine-treated group demonstrated a significantly greater improvement in the negative subscale of the Positive and Negative Syndrome Scale (PANSS) than the placebo-treated group (*p* < 0.001). The same clinical effect was observed for the PANSS total score (*p* < 0.001) and the general psychopathology subscale score (*p* = 0.002), but there was no significant difference in terms of reduction in the PANSS positive symptoms score between the two groups (*p* = 0.759) [25]. Subsequently, Matsuda et al. performed an update of their original meta-analysis by including data from the study by Rezaei et al. In 2013, they reported that memantine adjunctive therapy may ameliorate overall and negative symptoms in patients with schizophrenia [26]. Kishi et al. performed a meta-analysis for memantine add-on antipsychotic treatment in schizophrenia and reported in 2017 that memantine adjunctive therapy may be beneficial for treating psychopathological symptoms, particularly negative symptoms in schizophrenia patients. They also found that the size of the effect on negative symptoms may be greater in younger adult schizophrenia patients [27]. Di Iorio et al. conducted a systematic review. They reported in 2017 that increasing interest in memantine add-on treatment in patients with schizophrenia having negative and cognitive symptoms indicates that memantine may be a promising treatment option for schizophrenia [28]. The same systematic review also reported that memantine adjunctive therapy in patients with schizophrenia seems to ameliorate mainly the negative symptoms [28].

Furthermore, many articles have reported on memantine as adjunctive therapy in schizophrenia patients. Based on a randomized, double-blind, placebo-controlled 6-week trial, Fakhri et al. reported that memantine as an add-on treatment demonstrated significant improvement in the positive and negative PANSS subscale scores in patients compared to olanzapine alone (*p* < 0.001), and that female patients exhibited a better clinical response than male patients, especially in their positive PANSS score. The adverse events, including extrapyramidal symptoms, were not significantly different between the groups [29]. In 2017, Mazinani et al., based on a randomized, double-blind, placebo-controlled, 12-week trial, reported that memantine add-on treatment on risperidone significantly ameliorated negative (*p* = 0.003) and cognitive (*p* < 0.001) symptoms compared to risperidone alone in patients with schizophrenia [30]. In 2017, based on a randomized, double-blind, placebo-controlled, 12-week study in patients with schizophrenia, Omranifard et al. reported that memantine as an add-on treatment demonstrated significant improvement in positive symptoms (*p* = 0.028), negative symptoms (*p* = 0.004), general psychopathology (*p* < 0.001), depressive symptoms (*p* < 0.001), and total symptom severity (*p* < 0.001) compared to the placebo-treated group [31]. Hassanpour et al. conducted a double-blind, randomized, placebo-controlled, 8-week trial to evaluate the efficacy of memantine add-on administration compared to a standard regimen of antipsychotic treatment in patients with chronic schizophrenia. They reported in 2019 that memantine as an adjunct to the antipsychotic regimen demonstrated improvements in verbal memory (*p* = 0.01), working memory (*p* = 0.007), verbal fluency letter (*p* = 0.002), and verbal fluency total (*p* = 0.013) subscales of the Brief Assessment of Cognition Scale compared to the placebo-treated group [32]. Zheng et al. performed a meta-analysis of randomized, double-blind, placebo-controlled trials in patients with schizophrenia. They reported in 2018 that memantine as an add-on treatment appears to be effective in improving negative symptoms and neurocognitive performance [33]. Furthermore, on the basis of a systematic review and meta-analysis of double-blind, randomized, placebo-controlled trials, Zheng et al. reported in 2019 that memantine as adjunctive therapy appears to demonstrate significant improvement in negative symptoms and neurocognitive performance in patients with schizophrenia [34]. In a randomized, double-blind, placebo-controlled study, Schaefer et al. evaluated the clinical effects of memantine add-on treatment for 6 or 24 weeks compared to risperidone in patients with acute or chronic schizophrenia. In 2020, they reported that memantine as an add-on treatment for 6 weeks achieves a significant improvement in the areas of attention intensity (*p* = 0.005), verbal learning (*p* = 0.050), problem solving (*p* = 0.043), and flexibility (*p* = 0.049) in patients with acute schizophrenia, and that memantine as an add-on treatment for 12 weeks demonstrates significantly higher immediate memory and greater reduction in the PANSS total score than the placebo-treated group (*p* = 0.033 and *p* = 0.026, respectively) in patients with chronic schizophrenia [35]. Schaefer et al. emphasized that their study provides credence to the neuroprotective effects of memantine adjunctive treatment in improving cognitive function in patients with chronic schizophrenia [35].

It was reported that memantine may be a more promising option as an adjunct to clozapine therapy than non-clozapine antipsychotics, relying on a peculiar clozapine action at glutamatergic synapses [36,37]. de Lucena et al. performed a randomized, double-blind, placebo-controlled, 12-week trial of memantine add-on treatment to clozapine in patients with refractory schizophrenia. In 2009, they reported that memantine add-on treatment significantly improves (*p* < 0.01) the total Brief Psychiatric Rating Scale (BPRS) score (effect size = −2.75), positive symptoms score (effect size = −1.38), negative symptoms score (effect size = −3.33), the Clinical Global Impression (CGI) score (effect size = −1.56), and the Mini-Mental State Examination (MMSE) score (effect size = 1.32) compared to the placebo-treated group. Moreover, the extrapyramidal symptoms and weight gain were not significantly different between the two groups [38]. Veerman et al. performed a randomized, double-blind, placebo-controlled, 12-week crossover trial for memantine adjunctive therapy in patients with clozapine-treated refractory schizophrenia. In 2016, they reported that memantine significantly ameliorated PANSS negative subscale score (effect size = 0.29, *p* = 0.043) and the composite memory score (effect size = 0.30, *p* = 0.032), including verbal recognition memory and paired associates learning task scores on the Cambridge Neuropsychological Test Automated Battery (CANTAB), compared to placebo [39]. Veerman et al. conducted an open-label, 1-year extension study on memantine as an add-on treatment in patients with clozapine-treated refractory schizophrenia. They reported in 2017 that memantine add-on treatment to clozapine for 26 weeks achieves significant improvement in negative symptoms score of PANSS (effect size = 0.53, *p* < 0.001), positive symptoms score of PANSS (effect size = 0.50, *p* = 0.001), and PANSS total sores (effect size = 0.54, *p* < 0.001). In addition, it continues to demonstrate significant improvement in all the measures between 26 and 52 weeks that have effect sizes varying from 0.39 to 0.51. In their study, the CGI Severity Scale exhibited a moderate improvement, but not significantly, at 26 weeks (effect size = 0.36) and 52 weeks (effect size = 0.34), and there were no serious adverse effects [40].

Omranifard et al. performed a randomized, double-blind, controlled, 12-week clinical trial to evaluate the effect of memantine as adjunctive treatment on global function and quality of life in patients with schizophrenia. Enrolled patients were evaluated using the Global Assessment of Functioning (GAF) and quality of life scale (QLS) at baseline and every 4 weeks thereafter up to 12 weeks. There were no significant differences between the two groups in baseline GAF and QLS scores. However, GAF and QLS scores increased in both groups, with significantly higher scores in the memantine add-on group than the placebo group (*p* < 0.001 and *p* < 0.001, respectively) [41]. Omranifard et al. concluded that an add-on treatment with memantine significantly improved global functioning and quality of life of patients, and that if memantine can ameliorate cognitive and functional abilities in patients with schizophrenia, it may subsequently ameliorate patient function and quality of life [41].

Table 1 and Table 2 provide summaries of the meta-analysis studies and randomized, double-blind, placebo-controlled trials described above. Some evidence-based conclusions that memantine improves negative and cognitive symptoms, especially negative symptoms in schizophrenia patients, have been reached. Merritt et al. analyzed the nature of brain glutamate changes in schizophrenia by performing a meta-analysis of glutamate proton magnetic resonance spectroscopy study. They reported that schizophrenia is associated closely with an increase in glutamatergic neurotransmission in several limbic areas and indicates that compounds that can inhibit glutamatergic transmission may have potential to induce therapeutic efficacy [42]. This report seems to support the use of memantine as adjunctive therapy in schizophrenia.

## 4. Why is Memantine Free of the Severe Side Effects of NMDA Receptor Antagonists, such as PCP and Ketamine?

As mentioned in the section on the hypothesis of NMDA receptor hypofunction, the NMDA receptor antagonists like PCP and ketamine induced severe side effects, such as schizophrenia-like symptoms in humans [7,8,9,10]. However, the NMDA receptor antagonist memantine never causes such side effects at therapeutic doses in humans and was approved for Alzheimer’s disease due to its good safety profile [4]. It is increasingly being studied in a variety of non-dementia psychiatric disorders, such as schizophrenia, major depression, bipolar depression, and obsessive-compulsive disorder [43,44]. As explained below, there are numerous reports on memantine and other NMDA receptor antagonists that provide mode of actions on NMDA receptors.

### 4.1. Affinity for the PCP-Binding Site

The affinity and functional activity of NMDA receptor antagonists were investigated under the same assay conditions. The following NMDA receptor antagonists were tested: (+)MK-801 (dizocilpine), (S)-ketamine, (R)-ketamine, PCP, dexoxadrol, etoxadrol, memantine, amantadine, and dextromethorphan. A PCP binding assay using the radioligand [^3^H]MK-801 was performed with porcine-receptor membrane material [45]. (+)MK-801 showed very high affinity (Ki = 3.4 nM), dexoxadrol, etoxadrol, and PCP displayed moderate affinity (Ki = 25, 40, and 99 nM, respectively), and (S)-ketamine and memantine showed lower affinity (Ki = 440 and 740 nM, respectively). (R)-ketamine, dextromethorphan, and amantadine demonstrated remarkably low affinity (Ki = 1790, 3400, and 11,000 nM, respectively) [45]. In addition, the cytoprotective activities of the NMDA receptor antagonists were evaluated using cells expressing recombinant GluN1a/GluN2A or GluN1a/GluN2B receptors in a lactate dehydrogenase assay [45]. The cytoprotective activity data correlated with published IC_50_ values obtained based on two-electrode voltage clamp experiments (i.e., ion influx inhibition data), and a high correlation was observed between PCP affinity, ion flux inhibition, and cytoprotective activities [45]. The clinical performance of dexoxadrol, etoxadrol, (S)-ketamine, (R)-ketamine, amantadine, and dextromethorphan was reported as follows: Dexoxadrol and etoxadrol induced psychotomimetic effects similar to PCP in humans [46]. Intravenous injection of (S)-ketamine induced acute psychotic symptoms in healthy volunteers. In contrast, equimolar doses of (R)-ketamine did not induce psychotic symptoms [47]. A double-blind, crossover study for 18 weeks of amantadine add-on treatment on antipsychotics for the treatment of tardive dyskinesia indicated a low risk of amantadine in exacerbating psychosis and better management of tardive dyskinesia than placebo [48]. It was also reported that dextromethorphan is generally well-tolerated in humans, and the use of high doses over prolonged periods has been shown to be feasible [49]. From the above, as a point to be noted, the low affinity of memantine for the PCP binding site may be partly responsible for its good tolerability and safety profile in clinical practice. Parsons et al. reported that memantine, amantadine, and dextromethorphan, which have low affinities for NMDA receptors, exhibit obviously faster open-channel blocking/unblocking kinetics than NMDA receptor antagonists associated with severe psychotropic effects, such as PCP or (+)MK-801, which have high affinities for NMDA receptors, and unblocking kinetics are directly related to affinity, namely, lower affinity NMDA receptor antagonists have faster unblocking kinetics [50,51]. Heusler et al. also reported that memantine has faster unblocking kinetics than PCP and ketamine, similar to duloxetine, which is also a low-affinity antagonist of NMDA receptors, on recombinant human NMDA receptors [52] (it is described in more detail in the later Section 4.2 Mode of Fast Off-Rate and Section 4.3 Voltage Dependency).

### 4.2. Mode of Fast Off-Rate

In a review, Lipton discussed that the off-rate (i.e., the dissociation rate constant: K_off_) of an NMDA receptor antagonist from the ion channel may be a major factor to determine clinical tolerability. It is because an excessively slow off-rate causes the drug to accumulate in the channel, and the accumulated drug interferes with normal neurotransmission and causes the potential to induce unacceptable side effects. On the contrary, a too-fast off-rate may induce a relatively ineffective block in the channel to keep most of the normal transmission. The apparent affinity of a positively charged channel blocker is related to its off-rate (K_off_) divided by its on-rate (i.e., the associated rate constant: K_on_, K_d_ = K_off_/K_on_). The off-rate is an intrinsic property of the drug–receptor complex and is not related to drug concentration. The on-rate is related to the channel’s open probability and the drug’s diffusion rate and concentration. Memantine has a relatively fast off-rate (functionally, it corresponds to the faster unblocking kinetics as described above) compared to PCP and ketamine. Thus, the fast off-rate of memantine may be a major factor to minimize side effects in clinical practice [53]. Memantine, at therapeutic concentrations, enters the channel preferentially when it is pathologically activated under conditions of excessive glutamate exposure. The relatively fast off-rate prevents the drug from accumulating in the ion channels (i.e., the faster unblocking kinetics) and interfering with subsequent normal synaptic transmission to provide efficacy while displaying minimal side effects [50,51,53,54].

### 4.3. Voltage Dependency

**Figure 2:**
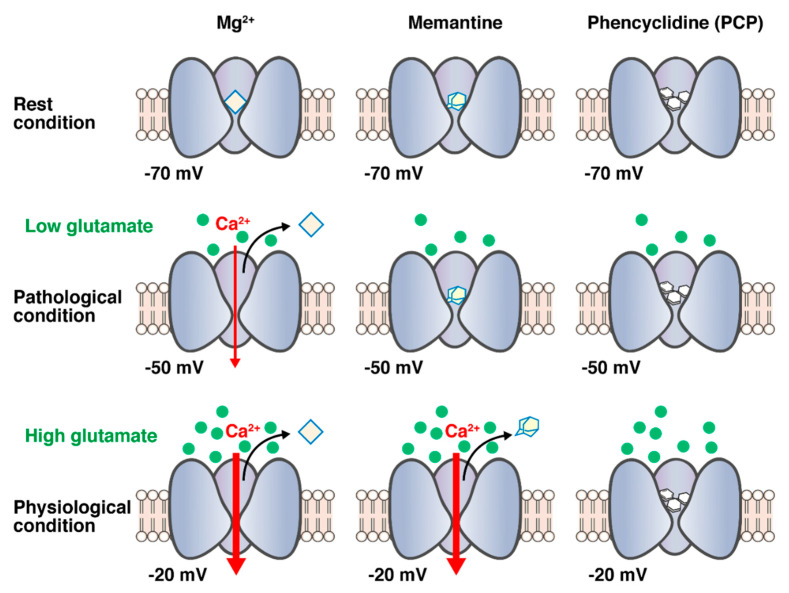
Schema of memantine for the hypothesis of blocking mode based on the combination of fast unblocking kinetics and strong functional voltage dependency. Under physiological conditions, the NMDA receptors are transiently stimulated by millimolar concentrations of glutamate following strong depolarization of the postsynaptic membrane (i.e., −20 mV) that rapidly removes Mg^2+^ from the binding site of the channel pore to relieve voltage-dependent blockade caused by Mg^2+^. However, the voltage dependency of Mg^2+^ is highly prominent so that it easily leaves the NMDA channel, even during moderate depolarization (i.e., −50 mV) under pathological conditions, and NMDA receptors are stimulated by low concentrations of glutamate for a much longer period. Memantine in contrast to PCP and (+)MK-801 seems to rapidly leave the NMDA channel during transient physiological activation by millimolar concentrations of synaptic glutamate, while it blocks the persistent activation by micromolar concentrations under the pathological conditions.

Parsons et al. reported that the blocking/unblocking kinetics of PCP and (+)MK-801 are too slow to allow them to leave the channel on depolarization, which may be attributed to the obviously weaker voltage dependency. As unblocking kinetics and voltage dependency are directly related to affinity, lower affinity NMDA receptor antagonists, such as memantine exhibit faster unblocking kinetics and obviously stronger voltage dependency. Parsons et al. also pointed out that, under physiological conditions, the NMDA receptors are transiently stimulated by millimolar concentrations of glutamate following strong depolarization of the postsynaptic membrane that rapidly removes Mg^2+^ from the binding site of the channel pore to relieve the voltage-dependent blockade caused by Mg^2+^. However, the voltage dependency of the divalent cation Mg^2+^ is highly prominent so that it easily leaves the NMDA channel even during moderate depolarization under pathological conditions, and NMDA receptors are stimulated by low concentrations of glutamate for a much longer period. Compounds with high affinities for NMDA receptors, such as PCP and (+)MK-801, have considerably slower unblocking kinetics than Mg^2+^ and are less functionally voltage-dependent. Therefore, the compounds cannot leave the channel within the time course of the normal excitatory postsynaptic potential mediated by NMDA receptors. Therefore, PCP and (+)MK-801 seem to block the physiological activation and pathological activation of NMDA receptors. Thus, the high-affinity and less voltage-dependent NMDA receptor antagonists, such as PCP and (+)MK-801, may induce unfavorable side effects. The voltage dependency of memantine is less functional than Mg^2+^, indicating that memantine can block only the activation of NMDA receptors during moderate depolarization under pathological conditions. Parsons et al. first proposed that the combination of fast off-rate kinetics and strong functional voltage dependency allows memantine to rapidly leave the NMDA channel during transient physiological activation by millimolar concentrations of synaptic glutamate, while it blocks the persistent activation by micromolar concentrations under the pathological conditions. Thus, memantine may display minimal side effects in clinical practice [50,51,54,55]. The schema of memantine for the hypothesis of blocking mode based on the combination of fast unblocking kinetics and strong functional voltage-dependency is represented in Figure 2.

Another theory to explain how NMDA receptor antagonists can leave the channel is partial trapping. High-affinity NMDA receptor antagonists, such as PCP and (+)MK-801, are completely trapped in the channel following the removal of the agonist, resulting in a complete block on the channel. On the contrary, the percentage of channels blocked by low to moderate affinity NMDA receptor antagonists decreases after agonist removal [54,55]. Interestingly, it was proposed based on electrophysiological studies that partial trapping may contribute to the good therapeutic profile of memantine, as about one-sixth of blocked channels (i.e., a percentage of channels of around 15% to 20%) would always be unblocked in the absence of an agonist and thereby be available for subsequent physiological activation [56]. The antagonism by memantine seems to act as a partial agonist with a relatively low intrinsic activity because it never causes 100% blockade of NMDA receptors, as mentioned by the Parsons’s group [55].

### 4.4. Uncompetitive Antagonist

A competitive antagonist binds to receptors at the same binding site to which the endogenous transmitter or exogenous agonist is bound. A competitive antagonist shows a parallel rightward shift of agonist-induced concentration–response curves with no observable change in the maximal response. On the other hand, a noncompetitive antagonist binds to an allosteric site on the receptors (at a site other than the agonist binding site) to prevent activation of the receptors. A noncompetitive antagonist shows a decrease in the maximal response of agonist-induced concentration–response curves, and in some cases, rightward shifts are produced. A noncompetitive antagonist is different from an uncompetitive antagonist. An uncompetitive antagonist is clarified as an inhibitor whose action depends on prior activation of the receptor by the agonist. This signifies that the same concentration of antagonist blocks higher concentrations of the agonist to a greater degree than lower concentrations of the agonist [53,57]. Chen et al. evaluated memantine for noncompetitive and/or uncompetitive components of antagonism based on whole-cell and single-channel recording experiments using rat retinal ganglion cells and demonstrated that memantine is a pure uncompetitive antagonist, in addition to its previously known mechanism of open-channel blocker [58,59]. In addition, Gilling et al. confirmed using human GluN1/GluN2A receptors expressed in HEK-293 cells that memantine acts as an uncompetitive antagonist [60]. The characteristic of being an uncompetitive antagonist for NMDA receptors leads to the potential clinical benefit of relatively sparing normal neurotransmission while blocking excessive channel activation by escalating levels of glutamate. Therefore, the good tolerability and safety profile of memantine in clinical practice may derive partially from the uncompetitive antagonist profile. Interestingly, it was reported in another similar case that ADCI {(±)-5-aminocarbonyl-10,11-dihydro-5H-dibenzo[*a*,*d*]cyclohepten-5,10-imine} is identified as an MK-801 analog with anticonvulsant activity and low toxicity in animals [61,62]. Voltage-clamp studies in cultured hippocampal neurons demonstrated that ADCI blocks NMDA receptor responses with its action as an uncompetitive NMDA receptor antagonist [61]. It was reported that ADCI shows potent anticonvulsant activities and a lack of PCP-like behavioral side effects in animals [62], supporting the theory that memantine, as an NMDA receptor uncompetitive antagonist, may be associated with minimal side effects in clinical practice. Song et al. studied how the GluN1-GluN2B NMDA receptor is blocked by MK-801 and memantine by combining crystallography with long-timescale molecular dynamics simulations. They reported in 2018 that MK-801 and memantine bind within the transmembrane domain vestibule of the ion channel and block ion conduction by physical occlusion of the permeation pathway and by promoting closure of the ion channel gate at the M3-helix-bundle crossing, yielding a closed–blocked state. MK-801 binds in two, two-fold-related poses, whereas memantine binds in a single predominant pose [63]. The differences in the tolerability and safety between MK-801 and memantine remain to determine whether or not they may be explained, at least partly, by the difference of binding profile of MK-801 and memantine in the study.

### 4.5. Preferential Inhibition of Extrasynaptic Receptors

It was reported that activation of synaptic or extrasynaptic NMDA receptors produces different signaling cascades and neuronal functions [64]. Based on the electrophysiological studies using rat autaptic hippocampal microcultures, Xia et al. (Lipton’s group) reported in 2010 that memantine at therapeutic concentrations (1–10 μM) preferentially blocks extrasynaptic rather than synaptic currents mediated by NMDA receptors in the same neuron and MK-801 blocks both synaptic and extrasynaptic NMDA receptors to the same degree [65]. Wu and Johnson also reported in 2015 that memantine selectively blocks extrasynaptic NMDA receptors that appear to contain GluN2C/2D subunits compared to synaptic NMDA receptors in rat substantia nigra dopamine neurons [66]. NMDA receptors located within the synapse were reported to facilitate cell survival pathways, whereas extrasynaptic NMDA receptors are likely to facilitate pathways leading to cell death [67]. Furthermore, treatment of transgenic mice (YAC128) with Huntington disease with memantine at low doses blocks extrasynaptic NMDA receptors instead of synaptic NMDA receptors and improves neuropathological and behavioral abnormalities [68]. On the basis of these findings, the fact that memantine possibly selectively blocks extrasynaptic NMDA receptors compared to synaptic NMDA receptors may explain its therapeutic effectiveness with minimal side effects in clinical practice. Savchenko et al. designed a hybrid nanodrug (AuM) that was larger than the synaptic cleft for attaching memantine via polymer linkers to a gold nanoparticle [69]. They demonstrated that AuM efficiently and selectively inhibited extrasynaptic NMDA receptors without inhibiting synaptic NMDA receptor transmission and that AuM exhibited neuroprotective properties both in vitro and ex vivo. In addition, AuM was able to inhibit dendritic spine loss caused by Aβ oligomers in organotypic hippocampal slices and was more effective than free memantine [69]. However, the observation that memantine inhibits NMDA receptors more effectively at higher glutamate concentrations based on uncompetitive antagonism does not explain preferential inhibition of extrasynaptic receptors, because extrasynaptic NMDA receptors are stimulated by much lower glutamate concentrations than synaptic NMDA receptors [70]. It is of interest whether or not some clinical variation of different NMDA receptor antagonists may be attributable to NMDA receptor subtype diversity. As described above, it was reported that activation of synaptic or extrasynaptic NMDA receptors produces different signaling cascades and neuronal functions [64]. GluN2C or GluN2D subunits were shown to exist predominantly in extrasynaptic regions [66]. The compound DQP-1105 is a selective NMDA receptor antagonist for GluN2C/2D subunits, which inhibits GluN2C- and GluN2D-containing receptors with IC_50_ values that are at least 50-fold lower than those for recombinant GluN2A-, GluN2B-, GluA1-, or GluK2-containing receptors [71]. DQP-1105 was more potent for blocking currents evoked by bath-applied NMDA compared to synaptic NMDA current, although MK-801 was equipotent for blocking currents evoked by bath-applied NMDA and synaptic NMDA receptors [66]. Thus, DQP-1105, as well as memantine, seems to have a potentiality to provide efficacy while displaying minimal side effects. Synthesis of other subunit-selective antagonists for NMDA receptors may accelerate progress in understanding the clinical roles of various NMDA receptor subunits.

In conclusion, it appears that memantine works as a low-affinity, fast off-rate, voltage-dependent, and uncompetitive antagonist with preferential inhibition of extrasynaptic receptors. The mode of actions of memantine for its NMDA receptor antagonist activity is represented in Table 3.

## 5. Can Memantine Ameliorate Abnormalities Caused by Other NMDA Receptor Antagonists or Caused by Other Manipulations?

Based on the hypothesis of NMDA receptor hypofunction in schizophrenia, it is of great interest whether or not memantine has ameliorating activity on the abnormalities caused by the hypofunction of NMDA receptors. There are several interesting reports related to this topic. Uribe et al. determined the effect of memantine on symptoms similar to schizophrenia in a rat model of ketamine-induced social withdrawal. They demonstrated that memantine treatment significantly ameliorated ketamine-induced social withdrawal, and that the amelioration was stronger than that of haloperidol by restoring the social interaction between rats that did not alter general motor activity [72]. These findings suggest that memantine may ameliorate social withdrawal by normalizing NMDA receptor hypofunction-induced abnormalities caused by ketamine. Diacylglycerol kinase (DGK) is an enzyme that regulates the balance between the two lipids diacylglycerol and phosphatidic acid and plays an important role as an intracellular signal transmitter [73]. DGKβ is a type 1 isozyme of the DGK family. DGKβ is closely related to formation of neurite spine, and DGKβ-knockout mice demonstrated behavioral disturbances, such as emotional, attentional, and cognitive impairment that may be attributed, at least in part, to the impairment of cortical spine formation [73]. It was reported that memantine significantly ameliorated the working memory in DGKβ-knockout mice in the Y-maze test. It was also demonstrated that the expression levels of the GluN2A and GluN2B subunits were increased in the prefrontal cortex, although they decreased in the hippocampus of DGKβ-knockout mice. The decreased expressions of GluN2A and GluN2B in the hippocampus may contribute to decreased LTP and spatial memory deficits in DGKβ-knockout mice [73]. Moreover, in rats neonatally administered PCP, it was reported that expression of GluN2A and GluN2B subunits in the prefrontal cortex was increased, but this was not seen in the hippocampus [74]. This neonatal PCP model in rats exhibited behavioral disturbances similar to symptoms of schizophrenia, including deficits of spatial working memory. Therefore, the abnormalities seen in rats neonatally treated with PCP may be derived, at least partly, from the increase in expression of GluN2A and GluN2B subunits in the prefrontal cortex [74]. Taking all the above facts into consideration, the altered expression levels of GluN2A and NR2B subunits especially in the prefrontal cortex in DGKβ-knockout mice might be related to the improvements in working memory brought about by memantine. However, in the same study, they were not able to confirm whether or not memantine would alter the expression levels of GluN2A and GluN2B in the prefrontal cortex and hippocampus of DGKβ-knockout mice [73].

Several microdialysis studies have demonstrated that systemic treatment with the NMDA receptor antagonists PCP, ketamine, and MK-801 increases glutamate release in the medial prefrontal cortex (mPFC), but local treatment with MK-801 into the mPFC does not affect glutamate release in the mPFC [75]. Fukuyama et al. demonstrated that a blockade of NMDA receptors in the mediodorsal thalamic nucleus (MDTN) caused by local administration of MK-801 into the MDTN increase the glutamate release in the mPFC [75]. Okada et al. obtained reproducible results that perfusion of MK-801 into the MDTN increased glutamate release in the mPFC without influencing that in the MDTN, suggesting that the main mechanism of increases in glutamate release in the mPFC induced by systemic treatment of NMDA receptor antagonists is produced by increased thalamocortical glutamatergic transmission outside of the mPFC [76]. Subsequently, Okada et al. demonstrated that the increase in glutamate release in the mPFC induced by systemic MK-801 treatment is inhibited by perfusions with muscimol, a GABA_A_ receptor agonist, into the MDNT but not by perfusion of muscimol into the mPFC. These results indicate that the increased glutamate release by systemic treatment of MK-801 is shown to follow activation of the thalamocortical glutamate pathway based on thalamic GABAergic disinhibition by NMDA receptor antagonism in the MDTN [76]. Interestingly, Okada et al. demonstrated that perfusion with memantine into the MDTN reduces MK-801-induced increases in glutamate release in the mPFC. Okada et al. suggested that the stimulatory effects of memantine on the astroglial system cysteine/glutamate antiporter may reduce MK-801-induced increases in glutamate release in the mPFC [76]. The possibility that the NMDA receptor blockade of memantine might be involved in the inhibitory action on MK-801-induced increases in glutamate release in the mPFC was not ruled out.

As mentioned above, anti-NMDA receptor encephalitis, which is caused by anti-NMDA receptor autoantibodies, is associated with severe psychiatric symptoms similar to schizophrenia. In anti-NMDA receptor encephalitis, antibodies to the NMDA receptors induce internalizations of the NMDA receptors, causing a progressive but reversible loss of surface NMDA receptors and leading to reduced function of NMDA receptors [21]. It was reported that memantine is effective in treating catatonia symptoms in schizophrenia and depression [77,78,79]. Interestingly, it was pointed out that catatonia may arise from NMDA receptor hyperfunction, although there is an apparent contradiction to catatonia symptoms seen in patients with anti-NMDA receptor encephalitis and to the NMDA receptor hypofunction hypothesis in schizophrenia [80]. Recently, Ramirez-Bermudez et al. reported in 2020 that a patient with anti-NMDA receptor encephalitis demonstrated amelioration in catatonia, alertness, attention, and agitation after receiving memantine for 2 days [81]. Memantine might ameliorate these clinical disturbances by normalizing NMDA receptor hypofunction-related abnormalities in anti-NMDA receptor encephalitis. Further clinical evaluation of memantine would be waiting in patients with anti-NMDA receptor encephalitis.

Reduction of the mismatch negativity (MMN) size, an event-related potential component evoked in response to unexpected stimuli, is perhaps the most robust neurophysiological finding in patients with schizophrenia. The sensitivity of MMN in predicting treatment response and clinical, cognitive, and psychosocial functions in schizophrenia and its ability to predict the transition to schizophrenia make it a potential neurophysiological biomarker for schizophrenia. Treatment with PCP and ketamine, the NMDA receptor antagonists, in healthy individuals produced a condition highly similar to schizophrenia, which also included MMN size reductions [82]. Swerdlow et al. were the first to evaluate the effects of memantine on prepulse inhibition (PPI) and MMN in patients with chronic psychotic disorders (CPDs). A double-blind crossover design trial, comparing (1) placebo versus memantine at 10 mg or (2) placebo versus memantine at 20 mg, included healthy individuals and patients diagnosed with schizophrenia, schizoaffective disorder, or depression types (i.e., CPDs). Memantine at the dose of 20 mg significantly increased PPI in individuals with CPDs and increased MMN across the groups. The effects on PPI were age-dependent and most pronounced in older patients, but effects on MMN were most pronounced in younger individuals. Memantine at 10 mg had no detectable effect or tended to reduce these measurements. Memantine demonstrated dose-dependent effects on preconscious, automatic measures of sensorimotor gating, and auditory sensory processing that are associated with enhanced cognition and function in patients with CPDs [83]. In 2020, Harms et al. reported that the most remarkable pharmacological finding for MMN made stronger the association between MMN and synaptic plasticity, as NMDA receptor antagonists reduced the MMN response. In addition, they reported that the association between NMDA receptor function and MMN is not as obvious as once thought because memantine, a low-affinity NMDA receptor antagonist, demonstrates increases in MMN [84].

Evidence indicates the relationship between a cortical glutamatergic hypofunction and disruption in auditory steady-state responses in the pathogenesis of schizophrenia [85]. Gamma oscillations and their regulation performed by NMDA receptors can be evaluated by their evoked power (γEP) and phase locking (γPL) in response to auditory steady-state stimulation. These responses of auditory steady-state can act as biomarkers for target engagement and early therapeutic effects [86]. Light et al. reported the effects of memantine (20 mg/day) on γEP and γPL in healthy individuals and patients with schizophrenia based on a double-blind, randomized, counterbalanced, cross-over design trial. Patients with schizophrenia demonstrated a significant decrease in γEP and γPL. Memantine significantly increased γEP and γPL in both healthy individuals and patients with schizophrenia. In patients with schizophrenia, significant correlations between age and effects of memantine were observed for γEP and γPL. Higher sensitivity to memantine for γEP and γPL was observed in younger patients with schizophrenia. Light et al. suggested that memantine acutely normalizes the cortical oscillatory dynamics related to NMDA receptor dysfunction in patients with schizophrenia [86].

A recent study report in 2020 suggested an association between cortical glutamatergic dysfunction and disruption in the dynamic balance of cortical excitation and inhibition (E/I) in the pathogenesis of schizophrenia. The study report also revealed that the abnormalities in the E/I balance in schizophrenia are normalized by acute treatment with memantine at 20 mg [87].

## 6. Can the NMDA Receptor Antagonist Activity of Memantine Be Exerted at Clinical Therapeutic Concentrations?

It needs to be determined whether or not memantine is an NMDA receptor antagonist with enough affinity to block NMDA receptors in the CNS at its therapeutic doses. It was reported that the free brain extracellular concentration of memantine is comparable to free serum and cerebrospinal fluid (CSF) concentrations in rats [88], and the CSF concentration of memantine is similar to serum concentration (a mean CSF/serum ratio = 0.52) in humans [89]. The therapeutically relevant serum concentration of memantine is thought to be around 1 μM [50]. Parsons et al. reported in a review in 1999 that, despite its relatively moderate affinity (Ki values of around 1 μM for the binding of ^3^H(+)-MK-801), memantine appears to be selective for the (+)-MK-801 site (i.e., PCP binding site) and does not inhibit the binding ligands for many other CNS receptors at concentrations of 10–100 μM [50]. From the above reports, memantine appears to exert its NMDA receptor antagonist activity in the CNS at therapeutic doses and the clinical efficacy of memantine is thought to derive primarily from its NMDA receptor antagonist activity [50].

After the report by Parsons et al. in 1999, it was reported that memantine has antagonist activity on 5-HT_3_ receptors and that 5-HT_3_ receptor antagonist activity might be related to improved negative symptoms in schizophrenia [90]. However, the IC_50_ values of memantine for serotonin-induced inward currents in HEK-293 cells expressing 5-HT_3A_ receptors and in the N1E-115 cell line with native 5-HT_3_ receptors were reported to be 2.2 and 2.29 μM, respectively [91]. The IC_50_ values were approximately two-fold higher compared to the therapeutically relevant serum concentrations (around 1 μM), suggesting that the effects of adjunctive administration of memantine on negative symptoms does not seem to derive mainly from its 5-HT_3_ receptor antagonist activity. Memantine has complex actions on α4β2, α7, and other nicotinic cholinergic receptors [90], and these actions may be related to the amelioration of cognitive impairments in schizophrenia [90,92]. However, it was reported that memantine inhibits α4β2 responses with an IC_50_ of 6.6 or 11.3 μM; these concentrations are much higher than the therapeutic concentrations (around 1 μM) [55]. In addition, the reported IC_50_ value of memantine at α7 receptors was 5 μM in human receptors [93]. The IC_50_ value of 5 μM was also higher than that of the therapeutic concentrations. Overall, the actions of memantine on α4β2 and α7 nicotinic cholinergic receptors may not be related to the ameliorating effects of memantine on cognitive impairments in schizophrenia. Seeman et al. reported that memantine binds the high-affinity state of D_2_ (D_2_^High^) receptors with a dissociation constant of 137 nM using human cloned dopamine D_2L_ receptors in Chinese hamster ovary (CHO) cells. Memantine as well as dopamine stimulated the incorporation of [^35^S]GTPγS into CHO cells containing human cloned dopamine D_2L_ receptors with a dissociation constant of 1200 nM. Memantine, at the concentrations between 200 and 2000 nM, directly acted on D_2_^High^ receptors to decrease prolactin release from isolated anterior pituitary cells [94]. The stimulating effect could be associated with the positive symptoms of schizophrenia, but it may contribute to the amelioration of negative symptoms [90]. However, it was reported that memantine does not decrease serum prolactin levels in humans [95], which suggests that memantine could not exhibit dopamine D_2_ receptor agonist activity in the human CNS. In conclusion, the effects of memantine as adjunctive therapy on negative symptoms and cognitive deficits in schizophrenia are thought to derive primarily, if not totally, from its NMDA receptor antagonist activity.

## 7. Conclusions

Memantine, an NMDA receptor antagonist approved for treating Alzheimer’s disease, has a good safety profile and is increasingly being studied for possible use in a variety of non-dementia psychiatric disorders. There is an abundance of data that support the hypothesis that NMDA receptor hypofunction contributes to the pathophysiology of schizophrenia. On the other hand, there are numerous clinical studies that have consistently reported that add-on treatment with memantine improves both negative symptoms and cognitive deficits, particularly the negative symptoms of schizophrenia, indicating that memantine as adjunctive therapy in schizophrenia helps to ameliorate negative symptoms and cognitive deficits. It remains unclear why memantine does not show undesirable CNS side effects in humans unlike the other NMDA receptor antagonists, such as PCP and ketamine. However, many reports have attempted to explain why this is so. Taking all the available evidence together, it would appear that memantine works as a low-affinity, fast off-rate, voltage-dependent, and uncompetitive antagonist with preferential inhibition of extrasynaptic NMDA receptors.

As detailed in Section 6 above, the CSF concentration of memantine is similar to serum concentration and the therapeutically relevant serum concentration of memantine is thought to be around 1 μM (20 mg/day as the clinical dose), which is similar to the IC_50_ values of memantine for PCP binding assay using the radioligand [^3^H](+)-MK-801. Therefore, the clinical dose of 20 mg/day appears to be able to exhibit NMDA receptor antagonist activity in the human CNS. Memantine was subsequently reported to act on some types of CNS receptors other than NMDA receptors. However, it is thought that the effects on those receptors could not be exerted fully in the clinical dose of memantine. Therefore, it is reasonable to assume that the effects of memantine as adjunctive therapy on negative symptoms and cognitive deficits in schizophrenia may derive primarily, if not totally, from its NMDA receptor antagonist activity.

## Figures and Tables

**Table 1:** Study reports for meta-analysis of randomized, double-blind, placebo-controlled trial.

Studies	Number of Trials	Patients	Drugs	Outcomes
Kishi et al. 2013 [24]	8 (*n* = 406)	Schizophrenia 85.5%, Bipolar disorder 14.5%	Memantine (3 trials, *n* = 186), Amantadine (5 trials, *n* = 220)	Not significant compared to placebo: overall symptoms (*p* = 0.31), positive symptoms (*p* = 0.44), negative symptoms (*p* = 0.16), and Clinical Global Impression Severity scale (*p* = 0.56). Memantine was favorable compared to placebo in Mini-Mental State Examination score in schizophrenia (*p* = 0.002).
Matsuda et al. (Kishi’s group) 2013 [26]	9 (*n* = 446)	Schizophrenia, Bipolar disorder	Memantine (4 trials, *n* = 226), Amantadine (5 trials, *n* = 220)	Not significant compared to placebo: overall symptoms (*p* = 0.08), positive symptoms (*p* = 0.43), and negative symptoms (*p* = 0.05). Individually, only memantine adjunctive therapy showed a trend towards superior efficacy of overall symptoms (*p* = 0.06) and negative symptoms (*p* = 0.06) compared to placebo.
Kishi et al. 2017 [27]	8 (*n* = 448)	Schizophrenia	Memantine	Significant compared to placebo: negative symptoms (*p* = 0.006), Positive and Negative Syndrome Scale general subscale (*p* = 0.002), and Mini-Mental Status Examination score (*p* < 0.0001). Not significant compared to placebo: overall (*p* = 0.06), positive (*p* = 0.07), and depressive symptoms (*p* = 0.326). Patient age was associated with memantine-associated amelioration of negative symptoms (slope = 0.171, *p* = 0.0206). The effect on negative symptoms may be greater in younger adult patients.
Zheng et al. 2018 [33]	8 (*n* = 452)	Schizophrenia	Memantine	Significant compared to placebo: Positive and Negative Syndrome Scale and Brief Psychiatric Rating Scale negative symptoms (*p* = 0.009) and Mini-Mental State Examination score (*p* < 0.00001). Not significant compared to placebo: total, positive and general symptoms (*p* = 0.06–0.60) and Clinical Global Impression Severity Scale (*p* = 0.78). There was no significant difference in the discontinuation rate (*p* = 0.31) and adverse drug reactions between the two groups.
Zheng et al. 2019 [34]	15 (*n* = 988)	Schizophrenia (9 trials, *n* = 512), Bipolar disorder (3 trials, *n* = 319), Major depressive disorder (3 trials, *n* = 157)	Memantine	In schizophrenia, significant compared to placebo: total psychopathology (*p* = 0.01), negative symptoms (*p* = 0.0003), and Mini-Mental State Examination score (*p* < 0.0001). Not significant compared to placebo: positive symptoms (*p* = 0.05) and general psychopathology (*p* = 0.14) in schizophrenia, depressive (*p* = 0.37) and manic (*p* = 0.09) symptoms in bipolar disorder, and depressive symptoms (*p* = 0.75) in major depressive disorder.

**Table 2:** Study reports for randomized, double-blind, placebo-controlled trial.

Studies	Patients/ Antipsychotics	Duration	Drug	Number	Outcomes
Rezaei et al. 2013 [25]	Schizophrenia (risperidone 6 mg/day)	8 weeks	Memantine (20 mg/day)	Memantine *n* = 20 Placebo *n* = 20	Significant compared to placebo: PANSS negative subscale score (*p* < 0.001), PANSS total score (*p* < 0.001), and general psychopathology subscale score (*p* = 0.002). Not significant compared to placebo: PANSS positive subscale score (*p* = 0.759). Changes in the Hamilton Depression Rating Scale score and the Extrapyramidal Symptom Rating Scale score and frequency of adverse effects did not differ between the 2 groups.
Fakhri et al. 2016 [29]	Schizophrenia (olanzapine 15–20 mg/day)	6 weeks	Memantine (week 1: 10 mg/day; weeks 2–6: 20 mg/day)	Memantine *n* = 30 Placebo *n* = 30	Significant compared to placebo: PANSS negative subscale score (*p* < 0.001) and PANSS positive subscale score (*p* < 0.001). Females showed significantly better response than males, especially in PANSS positive subscale score. No significant changes in extrapyramidal symptoms were observed.
Mazinani et al. 2017 [30]	Schizophrenia (risperidone 4–6 mg/day)	12 weeks	Memantine (titration weeks 1–4: 5 to 20 mg/day; weeks 5–12: 20 mg/day)	Memantine *n* = 23 Placebo *n* = 23	Significant compared to placebo: PANSS negative subscale score (*p* = 0.003) and Mini-Mental State Examination score (*p* < 0.001). Not significant compared to placebo: PANSS positive subscale score (*p* = 0.09) and general psychopathology score (*p* = 0.9).
Omranifard et al. 2017 [31]	Schizophrenia (atypical antipsychotic regimen)	12 weeks	Memantine (titration weeks 1–4: 5 to 20 mg/day; weeks 5–12: 20 mg/day)	Memantine *n* = 30 Placebo *n* = 30	Significant compared to placebo: PANSS negative subscale score (*p* = 0.004), PANSS positive subscale score (*p* = 0.028), general psychopathology score (*p* < 0.001), depressive symptom score (*p* < 0.001), and total symptom severity score (*p* < 0.001). No serious or severe adverse effects were reported during the study.
Hassanpour et al. 2019 [32]	Schizophrenia (antipsychotic regimen)	8 weeks	Memantine (week 1: 10 mg/day; weeks 2–8: 20 mg/day)	Memantine *n* = 20 Placebo *n* = 20	Significant compared to placebo: verbal memory (*p* = 0.01), working memory (*p* = 0.007), verbal fluency letter (*p* = 0.002), and verbal fluency total (*p* = 0.013) scores of the Brief Assessment of Cognition Scale. No improvement on psychotic symptoms was observed. There was no significant difference in the Abnormal Involuntary Movement Scale and Barnes Akathisia Rating Scale scores between the 2 groups.
Schaefer et al. 2020 [35]	Acute schizophrenia with predominant positive symptoms (risperidone 2–8 mg/day)	6 weeks	Memantine (20 mg/day)	Memantine *n* = 5 Placebo *n* = 6	Significant compared to placebo: attention intensity (*p* = 0.005), verbal learning (*p* = 0.050), problem solving (*p* = 0.043), and flexibility (*p* = 0.049). Not significant compared to placebo: PANSS total score and PANSS negative subscale score
	Chronic schizophrenia with negative symptoms (risperidone 2–8 mg/day)	24 weeks	Memantine (20 mg/day)	Memantine *n* = 7 Placebo *n* = 6	Significant compared to placebo: immediate memory (*p* = 0.033) and PANSS total score (*p* = 0.026). Not significant compared to placebo: PANSS negative subscale score.
de Lucena et al. 2009 [38]	Treatment- refractory schizophrenia (mean doses of clozapine memantine: 540 mg/day; placebo: 659 mg/day)	12 weeks	Memantine (20 mg/day)	Memantine *n* = 10 Placebo *n* = 11	Significant compared to placebo: Brief Psychiatric Rating Scale total score (effect size = −2.75, *p* = 0.001), positive symptoms score (effect size = −1.38, *p* = 0.007), negative symptoms score (effect size = −3.33, *p* = 0.001), Clinical Global Impression score (effect size = −1.56, *p* = 0.001), and the Mini-Mental State Examination score (effect size = 1.32, *p* = 0.005). No significant changes in extrapyramidal symptoms were observed.
Veerman et al. 2016 [39]	Treatment- refractory schizophrenia (clozapine dosage was remained as much unaltered as possible)	12 weeks	Memantine (week 1: 10 mg/day; weeks 2–12: 20 mg/day)	Memantine *n* = 26 Placebo *n* = 26 (each group has 52 cases based on two cross-overs)	This study was a 26-week single-center, double-blind, placebo-controlled crossover trial. The trial consisted of two crossover, 12-week treatment phases and a placebo wash-out period of 2 weeks in the 13th and 14th week to avoid carryover effects. Significant compared to placebo: PANSS negative subscale score (effect size = 0.29, *p* = 0.043) and memory composite (effect size = 0.30, *p* = 0.032), including verbal recognition memory and paired associates learning task scores on the Cambridge Neuropsychological Test Automated Battery. Not significant compared to placebo: PANSS positive subscale score (effect size = 0.15, *p* = 0.299) and PANSS total score (effect size = 0.19, *p* = 0.174). Side effects were mild and transient.
Omranifard et al. 2015 [41]	Schizophrenia (stable-dose atypical antipsychotic)	12 weeks	Memantine (titration weeks 1–4: 5 to 20 mg/day; weeks 5–12: 20 mg/day)	Memantine *n* = 32 Placebo *n* = 32	Global Assessment of Functioning scale and quality of life scale scores increased in both groups, with significantly higher scores in the memantine add-on group than the placebo group (*p* < 0.001 and *p* < 0.001, respectively). Memantine was well tolerated with no significant side effects.

PANSS: Positive and Negative Syndrome Scale.

**Table 3:** Hypothesis of mode of actions of memantine for its NMDA receptor antagonist activity.

Mode of Actions	Main References
Low affinity/fast off-rate	[45,50,51,53]
Voltage dependency/fast unblocking kinetics	[50,51,52,53,54,55]
Partial trapping	[54,55,56]
Uncompetitive antagonism/ agonist concentration dependency	[53,57,58,59,60]
Preferential inhibition of extrasynaptic receptors	[65,66,67,68,69,70]
